# Assessing, mitigating and controlling psychosocial hazards: a scoping review protocol to map the current state of knowledge

**DOI:** 10.1186/s13643-026-03170-5

**Published:** 2026-03-23

**Authors:** Yannick Metzler, Meike Heming, Mathias Diebig

**Affiliations:** 1 Department of Ergonomics, Leibniz Research Centre for Working Environment and Human Factors, Ardeystraße 67, Dortmund, 44139 Germany; 2 Institute of Occupational, Social, and Environmental Medicine, Centre for Health and Society, Medical Faculty and University Hospital Düsseldorf, Heinrich-Heine University Düsseldorf, Moorenstraße 5, Düsseldorf, 40225 Germany; 3 Department of Work and Organizational Psychology, Faculty I – Psychology, Trier University, Universitätsring 15, Trier, 54296 Germany

**Keywords:** Risk assessment, Psychosocial, Hazard, Risk, Assessment, Mitigation, Control

## Abstract

**Background:**

Psychosocial hazards are increasingly recognised as critical factors affecting employee health, safety, and well-being, arising from how work is designed, organised, and managed. In the European Union, employers are legally obliged to address these hazards as part of workplace risk assessment. While much is known about what constitutes a psychosocial hazard, the core risk management steps—assessment, mitigation, and control—are addressed only scarcely and inconsistently across studies and practices. This limits current understanding of how these steps contribute to reducing the risk of psychosocial hazards, hindering efforts to evaluate effectiveness and guide future research. This scoping review aims to map current knowledge related to these three foundational steps and explore their role in overall risk management.

**Methods:**

Guided by the Joanna Briggs Institute and PRISMA-ScR recommendations, this scoping review will be divided into three sub-reviews, each focusing on one step in psychosocial risk management: assessment, mitigation, and control. Comprehensive searches will be conducted in Scopus, Web of Science, PubMed, PSYNDEX, and APA PsycINFO for peer-reviewed publications since 2000 aligning with the EU legal framework. Grey literature sources will also be explored. Two independent reviewers will perform a multi-stage screening of titles, abstracts, and full texts using predefined inclusion and exclusion criteria. Data extraction will capture study characteristics, methods, and findings related to the three risk management steps. Narrative synthesis and tabular summaries will be used to present results, delineate knowledge gaps, and inform research and practice.

**Discussion:**

This review will provide a comprehensive overview of current knowledge on the assessment, mitigation, and control of psychosocial hazards, while identifying key research gaps. Its findings aim to inform policymakers, practitioners, and researchers on strategies to enhance worker well-being and support cohesive risk management. Although the EU focus enables an in-depth look at regulation-driven approaches, it may limit generalisability to non-EU contexts. Additionally, the scoping methodology, which prioritizes breadth over depth, will not allow for full evaluation of methodological quality or effectiveness. Nonetheless, the review will serve as a foundational map of the current state of psychosocial risk assessment and management, fostering dialogue and guiding future global research efforts.

**Systematic review registration:**

OSF number osf.io/t5cnu. 10.17605/OSF.IO/GNAMH.

## Introduction

Psychosocial hazards can significantly affect employees’ health and well-being [19, 21], which is why addressing them has become an essential part of occupational risk assessment. In risk science, hazards refer to work-related factors with the potential to cause harm, harm denotes the actual negative consequences of being exposed to hazards (e.g., mental or physical health impairment), and risk describes the probability that such harm will occur [17]. Risk management refers to the process of identifying these hazards, assessing the likelihood and severity of harm, and implementing measures to mitigate or eliminate the associated risks.

Psychosocial hazards represent a specific category of hazards, defined as work characteristics that may impair health and well-being, often arising from inadequate work design, organisation, or management [6]. Unlike more traditional hazards such as noise, chemicals, or physical strain, which are typically tangible, measurable, and linked to acute physical injury, psychosocial hazards are embedded in social and organisational work conditions and tend to exert their impact more subtly over time. They include many of the work characteristics central to occupational stress research, such as job demands, job control, social support, and role clarity [4].

Within the European regulatory framework, psychosocial hazards and risks are formally recognized under the Framework Directive 89/391/EEC, which obliges employers to systematically identify, assess, mitigate, and manage all work-related risks [8]. Accordingly, work analysis and work design as core competencies of work and organisational psychology, are increasingly embedded as tools to evaluate and improve work conditions through prevention-oriented risk management. Despite this regulatory integration, research has disproportionately focused on measurement and methodological development, such as questionnaire design [20, 23] or evaluations of policy frameworks [11, 12], leaving its actual potential to successfully mitigate psychosocial hazards relatively unexplored. Rick and Briner [18] provided one of the earliest comprehensive overviews of psychosocial risk management, but its brevity left important gaps. Since then, various reviews and articles have addressed specific issues, such as legal regulations [8, 16] or hazard conceptions and definitions [9, 10]. However, current scholarship tends to isolate certain steps or topics related to the process or tends to neglect the procedural interconnections essential to integrated risk management. In particular, the latter phases of the process—assessing, mitigating and controlling psychosocial hazards—remain insufficiently investigated, despite their critical function of turning analysis into action. Furthermore, interdisciplinary insights from across occupational science, which could help in understanding these later stages, probably remain unnoticed, either because of mismatched terminology or a lack of systematic summaries.

Addressing these gaps, this scoping review aims to comprehensively map the current state of knowledge concerning the steps of assessing, mitigating, and controlling psychosocial hazards since 2000 in the scope of the methodological approaches provided by the legal context of the EU. It will include recommendations for practice and policy and will provide a roadmap for future research. This scoping review will provide distinct findings for each of the three steps in the process, while also offering a combined perspective for contextual analysis. A scoping review is particularly suitable for this research because it facilitates the mapping of key concepts, methodologies, and knowledge across a broad domain. Unlike systematic reviews, which focus on answering specific questions on the basis of a narrow set of studies, a scoping review is more effective for exploring the breadth of knowledge and identifying research gaps [13].

## Objective

The general purpose of this scoping review is to develop an understanding of what is known about the steps of assessing, mitigating, and controlling psychosocial hazards in the scope of risk assessment. This scoping review aims to identify research gaps, inform policymakers about present challenges, and provide a roadmap for future research. Thus, our main research question is as follows:

What evidence-based findings exist regarding the steps of assessing, mitigating, and controlling psychosocial hazards in the scope of risk assessment in the European context after the year 2000?

This question serves as a general framework and will then be gradually applied to the specific steps. It was formulated through an iterative process of preliminary reading, exploratory searches and discussions within the review team, driven by a thorough examination of the research's objectives and the reasoning behind it. The review will focus on the following overall research questions:

1. What is known about these three steps: assessing, mitigating, and controlling psychosocial hazards; and which methods are applied in each step?
2. To what extent are the findings reported for each of the three steps methodologically robust?
3. What is reported about the strengths and weaknesses of the methods applied?
4. Are the three steps connected to the preceding and following steps, and how does this process impact overall risk management?

## Methods

### Scoping review method

This scoping review, as outlined, follows the framework methodology provided by Arksey and O'Malley [1] and Pollock et al. [15] and will be conducted in accordance with the current guidelines of the Joanna Briggs Institute [13, 14] and the Preferred Reporting Items for Systematic Reviews and Meta-Analyses extension for Scoping Reviews (PRISMA-ScR) [22]. The protocol has been registered in the Open Science Framework with the registration numbers osf.io/t5cnu and DOI 10.17605/OSF.IO/GNAMH.

### Eligibility criteria

#### Population

Risk assessment within the scope of European regulations is mandatory for employers. Hence studies focusing on employees and organisations across all occupational sectors and job roles, regardless of age, gender, or region, will be included. No specific sector or industry is exempt from this requirement.

#### Concept

The concept that will be studied is psychosocial risk management with a focus on the steps of assessing the risk of psychosocial hazards, mitigating this risk, and controlling it. This includes both theoretical frameworks and practical applications. All studies that either specifically research one of the respective steps, or provide knowledge on underlying concepts, theories, methods, practices, and empirical findings from the disciplines of occupational science will be included.

#### Context

The defining criteria for the population mandate the inclusion of all occupational contexts while excluding non-occupational contexts. The studies to be considered in this review stem from workplace settings, including any industry, company size, and geographical location. The review will focus primarily on literature targeting methodologies as applied in the European Union, where psychosocial risk assessments are influenced by relatively uniform legal frameworks under European directives. This does not imply that studies from countries outside of the EU will be automatically excluded but will be checked by the reviewers to determine whether the approach corresponds to approaches and methods as demanded by the EU regulatory Occupational Health and Safety framework. Studies published since 2000 will be included to ensure a contemporary understanding of the field. The following inclusion and exclusion criteria will be used to guide the search and applied when reviewing publications:

### Grey literature

In addition to peer‑reviewed publications, the search strategy will include grey literature exclusively from official governmental and statutory bodies, such as accident insurance institutions and occupational health and safety authorities. These sources were selected because they operate under a legal mandate to collect, analyse, and disseminate comprehensive workplace health and safety data via standardized reporting processes. Their outputs, while often not published in academic journals, provide authoritative, high‑quality empirical information that is directly relevant to the scope of this review and serves to complement and extend the evidence base identified in peer‑reviewed literature. The inclusion and exclusion criteria outlined in Table 1 apply equally to grey literature sources, with the exception of the peer-review criterion, which is not applicable by definition. The following sources for grey literature have been selected:

- European Foundation for the Improvement of Living and Working Conditions (Eurofound)
- European Agency for Safety and Health at Work (EU-OSHA)
- Partnership for European Research in Occupational Safety and Health (PEROSH)
- European Joint Research Centre (JRC)
- World Health Organization (WHO)
- Asia Pacific Academy for Psychosocial Factors at Work (APA-PFAW)
- International Commission on Occupational Health/Scientific Committee Work Organisation andPsychosocial Factors (ICOH-WOPS)
- International Labor organization (ILO)
- European Academy of Occupational Health Psychology (EAOHP)
- German Federal Institute for Occupational Safety and Health (BAuA)
- German Social Accident Insurance (DGUV)

**Table 1:** Inclusion and exclusion criteria for relevant material

Inclusion criteria	Exclusion criteria
• Publications after 2000 • Published in English or German • Employees as study population • Peer-reviewed publications • Corresponds to the context of the methodology and legal basis of the European Union • Relates to the context of assessment, mitigation and control steps of psychosocial risk management • Relates to occupational contexts	• Publication before 2000 • Not published in English or German • No employees as study population • Essays, opinions, commentaries, books, book chapters, secondary findings • Doctoral theses or conference abstracts when not listed and not peer reviewed • Does not correspond to the context of the methodology and legal basis of the European Union • Does not relate to the context of assessment, mitigation and control steps of psychosocial risk management • Does not relate to occupational contexts (e.g. studies on students, private life situations, pensioners, children, unemployed) • Research not applicable to work contexts (e.g. experimental studies unrelated to work) • Research solely providing descriptive summaries of current practice without analytical or evaluative component, legal requirements, or physical or non-psychosocial stressors and hazards

Moreover, three simplified search strings will be applied to Google Scholar, each targeting one of the three review steps (“assessing,” “mitigating,” and “controlling”) in combination with the key words “psychosocial hazard” and “risk assessment,” using the Publish or Perish software. For each query, the first 200 hits will be examined, following the approach outlined by Bramer et al. [3] and Haddaway et al. [7].

### Information sources and search strategy

The review team will develop the search strategy in collaboration with an experienced librarian. Additionally, two subject-matter experts will validate both the review’s purpose and the proposed search strategy. The general research question and corresponding overall aims already provide a segmentation according to the three steps of psychosocial risk management. Hence, this scoping review will essentially be segmented into three sub-reviews with different search strings each.

First, we will translate the research questions and identify key concepts and terminology to combine them with appropriate syntax, Boolean operators, and field codes. The JBI recommends a three-step search strategy for scoping reviews [2], beginning with a preliminary search in at least two databases, followed by analysing words and index terms in titles and abstracts used to describe the retrieved articles. A comprehensive search using all identified keywords and index terms across all included databases will be subsequently performed, with the third step specifically involving searching reference lists of identified articles, what is also described as backward search. Reference snowballing will be conducted during abstract screening to capture studies that may have been missed in the database searches. This approach ensures a comprehensive and systematic literature search, helping to identify a broad range of relevant studies for the scoping review.The sources to be searched include Scopus, Web of Science, PubMed, PSYNDEX, and APA psycINFO. Further information on publication types has already been provided in the inclusion and exclusion criteria.

In the following, we present a sample search string in PubMed for the ‘Assessment’ step. The search will be conducted during the year 2025. All strings are organized into logical blocks, (1) the psychosocial risk domain, i.e., generic terms capturing psychosocial risks and hazards equal to all steps of the risk assessment process; (2) the context, i.e., terms ensuring workplace and employee settings are covered; (3) the psychological/mental health dimension, i.e., terms linking hazards, stress and psychological outcomes, and (4) the step-specific methodology, i.e., terms specific to the respective phase. We deliberately opted for broader, controlled vocabulary terms in consultation with research librarians. These terms function as umbrella descriptors within database indexing systems, which by design encompass more specific subordinate concepts and concepts, for example, in the literature on work stress. This approach balances comprehensiveness with precision and avoids an unmanageable proliferation of search terms while still capturing literature on specific hazards through the hierarchical structure of database indexing.

("psychosocial risk management*"[All Fields] OR "psychosocial risk assessment*"[All Fields] OR "psychosocial risk*"[All Fields] OR "psychosocial hazard*"[All Fields].

("occupational stress"[All Fields] OR "occupational safety"[All Fields] OR "occupational health"[All Fields] OR "Occupational Health"[Mesh] OR "work safety"[All Fields] OR "OHS"[All Fields] OR "workload*"[All Fields] OR "worker*"[All Fields] OR "job"[All Fields] OR “workplace*”[All Fields] OR "employee*"[All Fields]).

("mental stress"[All Fields] OR "psychosocial risk*"[All Fields] OR "psychosocial hazard*"[All Fields] OR "psycho* stressor*"[All Fields] OR "psycho* stress"[All Fields] OR "Stress, Psychological"[Mesh] OR "psycho* health"[All Fields]).

("assessment"[All Fields] OR "analysis"[All Fields] OR "management"[All Fields] OR "risk evaluation"[All Fields] OR "matrix"[All Fields] OR "evaluation"[All Fields] OR "prioritization"[All Fields] OR "risk profile"[All Fields] OR "method"[All Fields] OR "monitoring"[All Fields] OR "threshold value"[All Fields] OR "reference value"[All Fields] OR "Psychometrics"[Mesh] OR "Psychometrics"[All Fields]) AND ("Metric"[All Fields] OR "benchmark"[All Fields] OR "Exposure"[All Fields] OR "cut$off"[All Fields] OR "analytical hierarchy process"[All Fields] OR "risk analysis"[All Fields] OR "probability"[All Fields] OR "limit value"[All Fields]).

### Study selection and screening

The study selection process will involve two independent reviewers at each stage of the screening process and for each step in risk management, on the basis of the inclusion and exclusion criteria outlined above:

1. Title and Abstract Screening: All records identified through the database search will undergo title and abstract screening. Studies that do not contain abstracts will be forwarded for full-text screening.
2. Full-Text Screening: The full texts of studies that met the inclusion criteria on the basis of title and abstract will be reviewed for final inclusion.

First, a pilot process will be conducted at both stages of screening, with two rounds of 10 records by two reviewers used for initial testing per step of risk management according to the data extraction form. Studies addressing multiple stages of psychosocial risk management will be coded multiple times, once for each relevant step, and assigned to the respective sub-reviews accordingly. Compatibility with EU methodological approaches will be assessed with reference to the PRIMA-EF framework [11], which provides an internationally recognized reference point for psychosocial risk management aligned with EU regulatory principles. Studies will be evaluated against the core procedural and methodological elements defined therein, rather than against specific national legislation. Disagreements regarding process stage assignment, EU compatibility, or any other screening decision will be resolved through discussion or by a third reviewer. All identified records suitable for review will be collated and uploaded into Rayyan.ai (Rayyan Systems Inc., Cambridge, MA, USA) to facilitate the organisation and removal of duplicates. The screening process will also use Rayyan.ai to streamline decision-making and ensure blind voting by reviewers. The results of the selection process will be presented in a PRISMA-ScR flow diagram to provide transparency regarding the included and excluded studies.

### Critical appraisal of included studies

In addition, although it is optional for scoping reviews, we will use an adjusted version of the Crowe Critical Appraisal Tool (V.1.4) [5] to assess the methodological robustness of the included studies, as displayed in Table 1 below. This is not done to rate the quality of studies as in systematic reviews but rather to obtain an impression about the overall quality of studies to map to which level the current state of knowledge is empirically sound. Specifically, the tool was adjusted by retaining only the four categories most relevant to methodological rigour (Design, Sampling, Data Collection, and Results); the remaining categories (Preliminaries, Introduction, Ethical Matters, and Discussion) were omitted as they primarily concern reporting and formal aspects rather than the methodological core of a study.

This will be carried out according to the same dual-review procedure as already outlined. This is particularly relevant given the heterogeneous nature of the literature in this field, which includes a substantial body of practice-oriented research from applied workplace settings where study designs and applied methods tend to vary considerably (Table 2).

**Table 2:** Critical appraisal tool [5] as adjusted for the scoping review

Category	Description of item (□ present; □ absent; □ not applicable)	Score (0–5)
**Design**		
Research design	1. Suitability of research design(s)	Design score
Intervention, Treatment, Exposure	1. Interventions(s)/treatment(s)/exposure(s) chosen and why 2. Precise details of the intervention(s)/treatment(s)/exposure(s) each group	
Outcome, Output, Predictor, Measure	1. Outcome(s)/output(s)/predictor(s)/measure(s) chosen and why 2. Clearly define outcome(s)/output(s)/predictor(s)/measure(s) 3. Outcome(s)/output(s)/predictor(s)/measure(s) valid and reliable	
Bias, etc	1. Potential bias, confounding variables 2. Group balance	
**Sampling**		
Sampling method	1. Sampling method(s) chosen and why 2. Suitability of sampling method	Sampling score
Sample size	1. Suitability of sample size	
Sampling protocol	1. Target/actual/sample population(s): description and suitability 2. Recruitment of participants/cases/groups	
**Data collection**		
Collection method	1. Collection method(s) chosen and why 2. Suitability of collection method(s)	Data collection score
Collection protocol	1. Include date(s), location(s), setting(s) 2. Method(s) to ensure/enhance quality of measurement/instrumentation	
**Results**		
Analysis, Integration, Interpretation	1. AII method(s) for primary outcome(s)/output(s)/predictor(s) chosen 2. Additional A.I.I. methods (e.g. subgroup analysis) chosen 3. Suitability of analysis/integration/interpretation method(s)	Results score
Essential analysis	1. Flow of participants/cases/groups through each stage of research 2. Demographic and other characteristics of participants/cases/groups 3. Response rate, non-participation/withdrawal/incomplete/lost data	
Outcome, Output, Predictor analysis	1. Summary of results □ and precision for each outcome/output/predictor/measure 2. Consideration of benefits/harms, unexpected results, problems/failures 3. Description of outlying data (e.g. diverse cases, adverse effects, minor themes)	

### Data extraction

A customized data extraction form is drafted by the review team in Microsoft Excel (Microsoft Corporation, Redmond, WA, USA) to extract the relevant information from eligible publications. The form is tested during the pilot process during study selection and screening. The form will be modified and revised as necessary during the extraction of the data based on reviewer’s consensus. Modifications will be detailed in the scoping review. Two independent reviewers will carry out data extraction per step of risk assessment. Discrepancies will be resolved by discussion or a third-party vote. Tables including descriptive and free-text summaries include the following data to recapitulate the evidence base:

- Authors
- DOI
- Year
- Study aims and research question
- Study design
- Study type
- Sample/Participant characteristics
- Sample size
- Sector (SIC Divisions)
- Step in risk assessment
- Underlying theoretical concept
- Methods used
- Outcomes researched
- Results
- Connection to overall risk management
- Country

### Data synthesis

The results will be synthesized into categories based on the steps in psychosocial risk assessment (assessment, mitigation, control). The data extracted will be presented in diagrammatic or tabular format in a way that aligns with the research questions, including descriptive statistics. The final data synthesis will also be narrative, summarizing the key findings and identifying gaps in the literature in line with the objective of this scoping review to map the current state of knowledge on the respective steps in psychosocial risk assessment. The synthesis approach may evolve as the review progresses and new findings emerge. In particular, the combined perspective across the three sub-reviews is specifically designed to address the procedural interconnections between the steps, directly corresponding to research question 4. This overarching synthesis will explicitly aim to identify how findings from the assessment, mitigation, and control steps relate to and inform one another.

### Strengths and limitations of the scoping review

This scoping review uniquely addresses the steps of assessing, mitigating and controlling psychosocial hazards that are often overlooked, ensuring a more holistic understanding of the current state of knowledge on psychosocial risk assessment. The predefined segmentation into three sub-reviews (assessment, mitigation, and control) facilitates a step-by-step analysis, enabling both in-depth explorations of each phase and synthesis across the risk management procedure. While focusing on the EU context clarifies the analytical scope, it may overlook relevant evidence from other legal and methodological contexts. However, studies from countries outside the EU are not automatically excluded but are reviewed for compatibility with EU methodological approaches; consequently, findings may hold relevance for other regions where similar approaches to psychosocial risk management are applied or under development. Additionally, restricting inclusion to English and German publications may exclude relevant studies from other linguistic contexts, potentially limiting the comprehensiveness of the evidence base. Nevertheless, as English serves as the working language of European Union institutions and the primary language for international scientific publishing, this restriction still provides broad access to EU-wide research output. However, as a scoping review, the emphasis on breadth over depth means methodological quality is not exhaustively appraised, tempering any conclusions about effectiveness in risk management.

## Conclusion

This scoping review aims to address existing gaps in the literature by providing a comprehensive map of the steps of assessing, mitigating and controlling psychosocial hazards in the scope of risk management. While previous reviews have focused on specific aspects, such as risk management or regulatory frameworks, no review has systematically examined these crucial steps. By synthesizing knowledge, methods, and impacts on overall risk management, this review will provide a clearer understanding of the field and offers a foundation for future research and practice in occupational health and safety.

## Data Availability

This protocol does not report data; any data generated during the full scoping review will be made available upon reasonable request, in supplementary materials, or publicly in the full review.
